# Biscuits Prepared with Enzymatically-Processed Soybean Meal Are Rich in Isoflavone Aglycones, Sensorially Well-Accepted and Stable during Storage for Six Months

**DOI:** 10.3390/molecules27227975

**Published:** 2022-11-17

**Authors:** Nathália Martins Bomfim Barreto, Diego Sandôra, Bernardo Ferreira Braz, Ricardo Erthal Santelli, Fabricio de Oliveira Silva, Mariana Monteiro, Daniel Perrone

**Affiliations:** 1 Laboratório de Bioquímica Nutricional e de Alimentos, Chemistry Institute, Federal University of Rio de Janeiro, Av. Athos da Silveira Ramos 149, CT, Bloco A, Sala 528A, Rio de Janeiro 21941-909, Brazil; 2 Laboratório de Desenvolvimento Analítico, Chemistry Institute, Federal University of Rio de Janeiro, Av. Athos da Silveira Ramos, 149, CT, Bloco A, Sala 518ª, Rio de Janeiro 21941-909, Brazil; 3 Laboratório de Desenvolvimento e Análise Sensorial de Alimentos, Faculty of Pharmacy, Federal University of Rio de Janeiro, Av. Carlos Chagas Filho, 373, CCS, Bloco A, 2nd floor, Sala 26, Rio de Janeiro 21941-902, Brazil; 4 Laboratório de Alimentos Funcionais, Nutrition Institute, Federal University of Rio de Janeiro, Av. Carlos Chagas Filho, 373, CCS, Bloco J, 2nd floor, Sala 16, Rio de Janeiro 21941-902, Brazil

**Keywords:** fermentation, microbiological stability, minerals, oligosaccharides, proximate composition

## Abstract

Soybean meal (SBM) is a co-product of the soybean oil industry that is rich in bioactive compounds, such as isoflavones. We aimed to study the effects of processing SBM by fermentation (*Saccharomyces cerevisiae*) (FSBM) and enzymatic hydrolysis (CelluMax C, a commercial cellulase) (ESBM) on its chemical composition, with emphasis on isoflavones. Fermentation increased protein content by 9%, ash content by 7%, dietary fiber by 11% and minerals by up to 38%, except for iron, which decreased by 26%. Fermentation completely removed oligosaccharides from SBM, while enzymatic processing decreased oligosaccharides by 45% in SBM. Both processes converted glycosylated isoflavones into the corresponding aglycones, the content of which increased by up to 7.7-fold. Biscuits containing SBM, FSBM and ESBM could be labeled as dietary sources of dietary fibers, potassium, phosphorous, calcium and zinc, as well as high in proteins, copper, iron, manganese and magnesium. While FSBM biscuits had lower sensory scores compared to SBM biscuits, ESBM biscuits had equivalent scores. During storage for 180 days at room temperature, the isoflavone profile of all biscuits remained stable. Moreover, storage did not impair microbiological and sensory qualities of any biscuits. Altogether, ESBM biscuits show great marketing potential.

## 1. Introduction

Soybean meal is a co-product of the soybean processing industry and is widely used in animal feed production. In addition to this main application, soybean meal can be used as a food ingredient to improve nutritional and functional value [1] and is the basis for a variety of soy protein products including soy flour, soy concentrate, soy isolates and textured soy protein [2]. Soybean meal presents high protein, fiber and minerals contents, as well as other minor components, such as trypsin inhibitors, phytic acid, saponins and phenolic compounds [3,4]. Among the phenolic compounds, isoflavones are the most abundant class and can be found as aglycones or glycosides, especially malonylglycosides and β-glycosides [3].

The high consumption of soy and its derivatives has been considered one of the reasons for the low incidence of breast and prostate cancer in Asian populations [5]. In addition, studies show that the consumption of these foods is associated with a lower risk of developing cardiovascular diseases, improved bone health and prevention of menopausal symptoms, among others beneficial effects [5,6]. Most of these outcomes are related to the presence of isoflavones, which possess antioxidant and anti-inflammatory activities, as well as estrogen receptor affinity [6,7,8].

Bioactivity of isoflavones is highly dependent on their bioavailability. Aglycones have higher bioavailability compared to glycosylated forms [9,10]. In soybeans, the conversion of glycosylated forms to aglycones may occur as a consequence of germination [11], enzymatic hydrolysis [12] and, most commonly, fermentation [13,14,15]. The latter process provides desirable changes in the flavor and texture of fermented soy-based foods, such as tempeh, dou-chi, miso and natto, as amino acids, peptides, fatty acids and sugars, which impact physical—chemical and sensory aspects, are generated [16,17]. However, in soy-based products that are not traditionally fermented, these compounds may be perceived as off-flavors, negatively affecting their acceptance, as observed by Silva et al. [1]. These authors reported lower consumer acceptance for biscuits made with fermented soybean meal in comparison to biscuits made with non-fermented soybean meal. Therefore, even though biscuits made with fermented soybean meal had high contents of aglycone isoflavones, and thus high bioavailability [10], they were not commercially viable.

To deal with this issue, enzymatic processing could be used as an alternative to yield biscuits with high contents of aglycones and, at the same time, give good sensory acceptance. Enzymatic processing of soybean meal has already been employed to improve the digestibility of nutrients in animal feed by reducing anti-nutritional factors and allergenic proteins. The main enzymes used are proteases, such as alkalase, and carbohydrases, such as pectinase, xylanase, cellulase and α-galactosidase, among others [18]. Jacobsen et al. [19] reported reduction in phytic acid and saponins in soybean meal processed with an enzymatic extract produced by solid state fermentation of *Aspergillus niger*. Kano et al. [20] observed the conversion of isoflavone glycosides to aglycones in soy milk upon treatment with β-glucosidase. Therefore, the aim of this study was to compare the effect of fermentation and enzymatic bioprocessing on nutritional composition, isoflavone profile and sensory acceptance of biscuits. Additionally, we evaluated their chemical, sensory and microbiological stabilities during storage for 180 days.

## 2. Results and Discussion

### 2.1. Fermentation and Enzymatic Processing of Soybean Meal Decreases Oligosaccharides Contents and Converts Isoflavones to Aglycones

Fermentation of SBM caused increases in protein content (9%), ash content (7%) and total dietary fiber (11%). These results are probably not a consequence of yeast anabolism, but rather a relative change caused by the catabolism of total carbohydrates and lipids, the contents of which reduced by 58% and 13%, respectively (Table 1). Increases in protein contents of between 10% and 14% after fermentation of soybean meal with different microorganisms has been reported in the literature [21,22]. A 50% dietary fiber increase due to fermentation of soybean meal with baker’s yeast has been observed in previous work from our group [1]. On the other hand, fermentation with baker’s yeast caused decreases in the content of crude fiber of soybean meal (49%) [22] and okara (from 27% to 40%) [23], which could be attributed to the secretion of complex polysaccharide-degrading enzymes. These conflicting results could be explained by the different methods of analysis (crude fiber and enzymatic-gravimetric total dietary fiber) used in these studies. Concerning enzymatic processing, the proximate composition of ESBM was very similar to that of SBM (Table 1).

The content of minerals in all samples was similar to those reported for soybean meal samples [4]. Fermentation caused increases of up to 38% in the content of all minerals, with the exception of iron, where content decreased by 26% (Table 1). It has been reported that the fermentation process may increase the mineral content of legumes due to the degradation of macromolecules and loss of dry matter [24]. The decrease in iron could be attributed to its uptake by *S. cerevisiae* through siderophore transport [25]. On the other hand, Li et al. (2020) [26] did not observe differences in iron, calcium, zinc and potassium content in whole soybean flour after fermentation with *Lactobacillus casei.* As observed for the proximate composition, the ESBM mineral profile was very similar to that of SBM (Table 1).

The profile of oligosaccharides in SBM was in accordance with the literature [21]. Fermentation led to the complete removal of oligosaccharides, which could no longer be detected in this sample, probably due to their hydrolysis and consumption by baker’s yeast; α-galactosidase [27] and invertase activities [28] have already been reported in the literature. The reduction in stachyose and raffinose due to fermentation of soybean meal was also observed by Tudor et al. (2013) [29] using *S. cerevisiae* and by Chen et al. (2010) [21] using *Aspergillus* and *Lactobacillus*. Singh and Vij (2018) [30] observed decreases in the contents of oligosaccharides during fermentation of soy milk with *Lactobacillus*. Enzymatic processing of soybean meal led to decreases in the contents of sucrose (47%), raffinose (48%) and stachyose (41%), suggesting that CelluMax C had α-galactosidase and invertase activities, in addition to cellulase and xylanase. Due to the lower content of oligosaccharides, the use of FSBM and ESBM in the food industry would yield products that would cause less intestinal discomfort than those produced with non-processed soybean meal [5].

SBM presented approximately 165 mg/100 g of total isoflavones (Table 2), with β-glycosides as the major subclass (80% of total isoflavones), followed by aglycones (10%), acetylglycosides (6%) and malonylglycosides (4%) subclasses (Figure S1). This profile was similar to that reported by other studies with soybean meal and defatted soybean flour [1,13]. Both fermentation and enzymatic processing of SBM caused the conversion of β-glycosides, malonylglycosides and acetylglycosides into the corresponding aglycones, the contents of which increased 7.7- and 6.1-fold, respectively. This profile change due to fermentation was also observed when soybean meal was treated with baker’s yeast [1] and when soybean flour was treated with *Aspergillus oryzae* or *Monascus purpureus* [13]. The use of enzymatic processing to increase aglycone contents in soy-based foods also has been described in the literature. Kano et al. (2006) [20] observed that treatment of soy milk with isolated β-glucosidase and fermentation with *Bifidobacterium breve* and *Lactobacillus mali* led to similar changes in the profile of isoflavones. Moreover, Queirós et al. [12] reported that the treatment of soybean flour with an isolated cellulase caused the conversion of daidzin and genistin to their corresponding aglycones. While enzymatic processing of SBM with CelluMax C is less efficient than fermentation with baker’s yeast in converting isoflavones to aglycones, the latter process yields other metabolites, such as organic acids, which could act as off-flavors and negatively impact the sensory acceptance of foods produced with FSBM.

### 2.2. Enzymatic Processing of Soybean Meal Yields Biscuits with High Nutritional Value, Rich in Aglycone Isoflavones and Good Sensory Acceptance

The proximate composition of SBM, FSBM and ESBM biscuits (Table S1) was similar to that reported in previous work from our group concerning soybean meal biscuits [1]. The consumption of one portion (30 g) of any of the soybean meal biscuits would represent between 13% and 18% of the dietary recommended intake (DRI) for protein and between 11% and 24% of the DRI for dietary fiber (Table 3). Considering the guidelines on nutrition labelling of the *Codex Alimentarius* (2017), all samples could be considered as high in proteins and as sources of dietary fibers. It should be highlighted that soy proteins are among the plant proteins with the highest biological values. Considering that the recommendation ranges from 25 to 30 g/day, the consumption of ~two portions of soybean meal biscuits could contribute to reaching the recommended intake of this component. The dietary fibers in soybeans are mostly insoluble, such as cellulose and hemicellulose, and their intake is associated with the reduction in intestinal transit time, prevention of constipation and improvement in glucose and cholesterol metabolism [31]. Considering that the chronic consumption of 100 g of biscuits containing 27.5 g of soybeans dietary fibers reduced body weight, waist circumference, total cholesterol and LDL-cholesterol in overweight adults [32], one could hypothesize that the daily consumption of our soybean meal biscuits could lead to these beneficial health outcomes.

Regarding minerals, the consumption of one portion of SBM biscuits would represent between 4% (sodium, for both men and women) and 26% (iron, for men) of the DRI (Table 3). Even though fermentation of soybean meal led to a decrease in the contribution to the DRI of iron (16% for men), it should be noted that its bioavailability might be favored due to the concomitant decrease in the content of anti-nutrients, such as phytate, oxalate and tannins, as observed in the literature [24]. In terms of product labeling, all biscuits could be marketed as dietary sources of potassium, phosphorous, calcium and zinc, as well as high in copper, iron, manganese and magnesium.

The total contents of isoflavones in SBM, FSBM and ESBM biscuits were 99 mg/100 g, 76 mg/100 g and 65 mg/100 g, respectively (Table 4). The FSBM biscuits had higher contents of aglycone equivalents (75 mg/100 g) in comparison to SBM (65 mg/100 g) and ESBM (62 mg/100 g) biscuits. Each biscuit (weighting approximately 5.5 g) contained, on average, 4.2 mg of total isoflavones, and their consumption could positively contribute to human health due to the functional properties of isoflavones, such as antioxidant and anti-inflammatory activities [6,7]. According to Messina et al. [33], daily consumption of 25 mg to 50 mg of isoflavones could reduce the risk of prostate and breast cancers. This dose could be achieved by the consumption of one to two portions (30 g) of our soybean meal biscuits. It should also be noted that fermentation and enzymatic processing of SBM may improve the health benefits associated with the consumption of biscuits, since isoflavone aglycones are more bioavailable than glycosylated forms [14,20]. Recently, we have reported that the consumption of FSBM biscuits led to a higher urinary excretion of isoflavones and their metabolites when compared to SBM biscuits [10].

Fermentation is one of the major processes used in the production of soy foods in Asian countries, such as tempeh, dou-chi, miso and natto. In addition to converting glycosylated isoflavones to their aglycones, possibly favoring their bioavailability and bioactivity, fermentation also modifies the sensory attributes of soy products [15]. We have recently reported that FSBM biscuits had a lower overall sensory acceptance than SBM biscuits, mainly due to a 45% decrease in taste scores, which was caused by a sour flavor. Fermentation is a complex bioprocess with modification in texture, taste and aroma of the final product due to the formation of many compounds such as peptides, sugars, amino acids, fatty acids, alcohols, ketones, organic acids and aldehydes [16]. Once again, we have observed that FSBM biscuits had lower scores for overall acceptance, taste, aroma and purchase intent in comparison to SBM biscuits (Table 5). In this sense, in the present work, enzymatic processing was used in an attempt to minimize changes in the sensory attributes of soybean meal biscuits. In fact, ESBM biscuits had equivalent scores in comparison to SBM biscuits, except for aroma, which showed a slight decrease of 16%. Nevertheless, this decrease did not impair the purchase intent of ESBM biscuits.

### 2.3. Soybean Biscuits Showed Isoflavones, Sensory and Microbiological Stability after 180 Days of Storage

To investigate the shelf-life of soybean meal biscuits, samples were stored at room temperature (25.7 ± 2.2 °C), vacuum packed and protected from light for 180 days. At day zero, water activity ranged from 0.28 to 0.45 and increased during storage for SBM and ESBM biscuits. Nevertheless, after 180 days of storage, water activity remained below 0.45 for all biscuits, lower than the critical value required for microbial spoilage (<0.60) [34]. In fact, all biscuits were microbiologically adequate at day zero according to Brazilian legislation and remained safe after 180 days of storage (Table S2).

**Figure 1:**
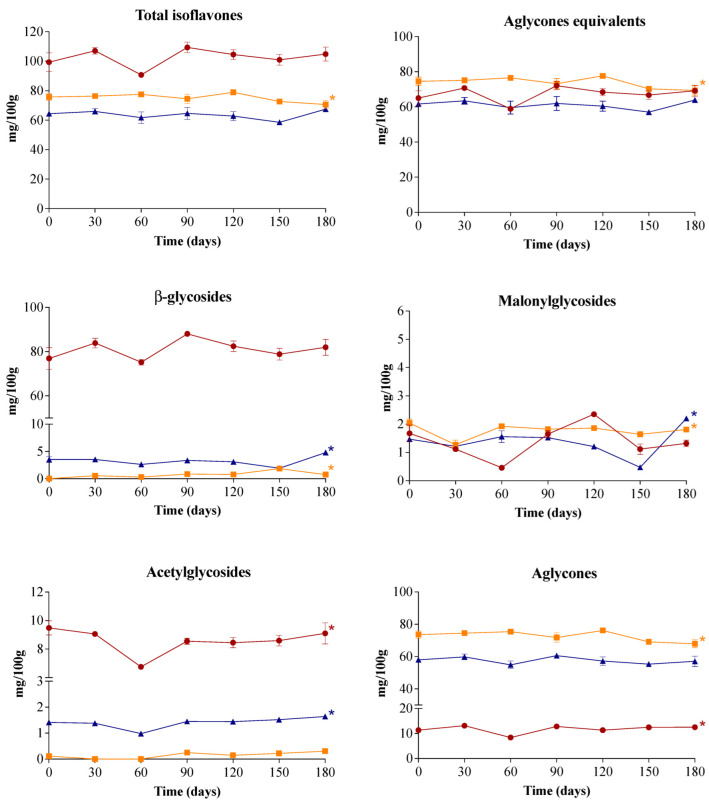
Contents of isoflavones and their subclasses in soybean meal (SBM) (●), fermented soybean meal (FSBM) (■) and enzymatically-processed soybean meal (ESBM) (▲) biscuits during storage at 25 °C for 180 days. Data expressed as mean ± standard deviation (*n* = 3) on dry weight basis. Asterisks indicate significant differences between initial (day 0) and final (day 180) storage times (ANOVA followed by Dunnett’s post hoc test; *p* < 0.05).

For all biscuits, the isoflavone profile remained stable during storage (Figure 1). The content of total isoflavones and aglycones equivalents of SBM and ESBM biscuits did not change after 180 days of storage, while FSBM biscuits showed a slight reduction of 7%. Silva and Perrone [3] also observed isoflavone stability of dry extracts obtained from soybean meal stored for 180 days at room temperature. On the other hand, various studies have reported the loss of soy isoflavones in foods, which apparently follows first order degradation kinetics [35]. These studies, however, were conducted in foods of very high values of water activity (i.e., tofu and soymilk) and/or at temperatures higher than 27 °C.

After 180 days of storage, sensory attribute scores of SBM and FSBM biscuits did not change (Table 5). For ESBM biscuits, aroma and texture scores increased by 19% and 13%, respectively, after storage. The change in the aroma score could be attributed to volatilization of off-flavors formed during enzymatic processing during storage. It should also be noted that storage did not impair the purchase intent of either biscuit.

## 3. Materials and Methods

### 3.1. Standards and Chemicals

Standard solutions of minerals were purchased from Quimlab Química & Metrologia^®^ (São Paulo, Brazil). Commercial standards of stachyose and sucrose were purchased from Sigma (St. Louis, MO, USA), raffinose from Merck (Darmstadt, Germany) and daidzin, glycitin, genistin, daidzein, glycitein and genistein from Apin Chemicals Limited^®^ (Abingdon, UK). A commercial food grade fungal cellulase enzyme (CelluMax C) was donated from Prozyn (São Paulo, Brazil). All solvents were HPLC grade from Tedia (Fairfield, OH, USA) and HPLC grade water (Milli-Q system, Millipore, Bedford, MA, USA) was used throughout the experiments.

### 3.2. Soybean Meal Bioprocessing

A soybean meal (SBM) sample was provided complimentary by Cargill Brazil, a soybean oil crush industry, and subjected to either fermentation or enzymatic processing. Fermentation was performed as described by Silva et al. [1]. SBM was homogenized with water (SBM:water, 1:2, *w*/*v*) and baker’s yeast (*Saccharomyces cerevisiae*) (baker’s yeast:SBM, 1:150, *w*/*w*) was added. The mixture was incubated at 40 °C for 48 h and the fermented soybean meal (FSBM) was dried in an oven at 55 °C for 24 h, milled and stored at −20 °C. Enzymatic processing was performed using CelluMax C, which, in addition to cellulase activity (872.6 U/g), also showed xylanase activity (182.3 U/g). SBM was homogenized with water (SBM:water, 1:3, *w*/*v*), CelluMax C (CelluMax C:SBM, 0.1:100, *w*/*w*, equivalent to 87.3 U of cellulase/100 g of SBM) was added and the mixture was incubated at 25 °C with agitation (140 rpm) for 30 min. Enzymatically-processed soybean meal (ESBM) was dried in an oven at 55 °C for 24 h, milled and stored at −20 °C.

### 3.3. Biscuit Preparation

Biscuits were prepared according to Silva et al. (2018) [1] using either SBM, FSBM or ESBM, in addition to sugar, salt, wheat flour, baking powder and an artificial sweetener (mixture of acesulfame K and sucralose) (Table S3). Dry ingredients were mixed thoroughly and after the addition of water, vanilla extract and margarine, the dough was homogenized and rolled out into disks of 4.5 cm diameter. Biscuits were baked in an oven at 180 °C for 15 min, cooled to room temperature and stored at −20 °C until chemical analyses.

### 3.4. Stability Test of Biscuits

SBM, FSBM and ESBM biscuits were stored at room temperature, vacuum packed and protected from light for 180 days. Every 30 days, biscuits were ground and analyzed for moisture [36], water activity at 25 °C using the LabMaster-aw analyzer (Novasina, Pfáf-fikon, Switzerland) and isoflavone profile, as described in Section 3.8. Before and after 180 days of storage, biscuits were submitted to microbiological (Section 3.9) and sensory analyses (Section 3.10).

### 3.5. Proximate Composition of Soybean Meals and Biscuits

SBM, FSBM, ESBM and their corresponding biscuits were analyzed in triplicate for moisture, ash, protein, lipid and total dietary fiber, and total carbohydrate was determined by difference, according to the official methods of the AOAC [36].

### 3.6. Mineral Profile of Soybean Meals and Biscuits

SBM, FSBM, ESBM and their corresponding biscuits were digested in duplicate using the USEPA 3051A method [37]. Briefly, 0.5 g aliquots of previously dried and ground samples were mixed in 10 mL of concentrated nitric acid and gradually heated in a microwave digestion oven (SpeedWave 4, Berghof, Eningen, Germany).

Calcium, copper, iron, phosphorus, magnesium, manganese, potassium, sodium and zinc contents were determined by ICP OES [38]. The spectrometer had radial vision (Horiba Jobin Yvon, Ultima 2, Longjumeau, France), equipped with a cyclonic spray chamber and a MiraMist type parallel flow nebulizer (Mira Mist EC, Burgener Research Inc., Mississauga, ON, Canada), an AS 421 automatic sampler and Analyst 5.4 operational software for data acquisition. The operating conditions were 1200 W of incident power, 12 L/min of plasma gas flow rate, 0.2 L/min of coating gas flow rate, 0.02 L/min of nebulization gas flow rate, 1 bar of nebulizer pressure, 1.0 mL/min of sample introduction flow rate, 1 s of integration time and high resolution. The following wavelengths were used for each element: Fe (259.940 nm), K (766.490 nm), Mn (257.610 nm), Na (588.995 nm), P (214.914 nm), Ca (396.847 nm), Zn (213.856 nm), Cu (324.750 nm), and Mg (279.553 nm). Quantification was performed by interpolation using an analytical curve with four standard solutions for calibration. Results were expressed as mg of compound per 100 g on a dry weight basis (dwb).

### 3.7. Isoflavone Profile of Soybean Meals and Biscuits

Extraction of isoflavones in SBM, FSBM, ESBM and their corresponding biscuits was performed in triplicate, as described by Silva and Perrone [3]. Briefly, 2 g of ground sample was extracted with 10 mL of a ternary solvent mixture (water:ethanol:ethyl acetate, 40:40:20, *v*/*v*/*v*) in a vortex for 2 min. After centrifugation for 10 min at 1000× *g*, the supernatant was collected in a volumetric flask and the residue was re-extracted twice. Prior to injection, extracts were filtered through a 0.45 μm PTFE filter unit.

Isoflavones were analyzed by LC-DAD-MS, as described by Fonseca et al. [39]. The HPLC system (Shimadzu, Kyoto, Japan) was composed of two parallel LC-20AD binary pumps, an SPD-M20A diode array detector (DAD), a CBM-20A control system, a DGU-20A5 degasser and a SIL-20AHT automatic injector, which was coupled to a LCMS-2020 mass spectrometer with an ESI ion source. Chromatographic separation was achieved using a Kinetex^®^ C18 column (50 × 2.1 mm, 5 μm, 100 Å, Phenomenex Torrence, CA, USA) (Figure S2). The LC two-phase mobile system consisted of a gradient of water (eluent A) and acetonitrile (eluent B), both added with 0.3% formic acid, with a constant flow rate of 0.6 mL/min. Prior to injection, the column was equilibrated with 10% solvent B. After injection of 20 μL of sample, this proportion was modified to 17% solvent B after 6 min, 21% solvent B after 12 min and 10% solvent B after 15 min. Between injections, 5 min intervals were used to re-equilibrate the column with 10% solvent B. Isoflavones were monitored by DAD at 254 nm and by MS using positive ionization, with a nebulizer gas (N_2_) flow of 3.0 L/min, operated in the single ion monitoring (SIM) mode to detect pseudomolecular ions. Data were acquired by LabSolutions software (Shimadzu Corporation^®^, version 5.82, 2015).

Identification of compounds was performed by comparison with retention time and molecular weight of the respective standard. Identification of compounds for which there were no commercial standards available were performed by the pseudomolecular ion in the MS. Quantification was performed by external standardization. The contents of malonylglycosides and acetylglycosides isoflavones were determined from the calibration curve of the corresponding β-glycoside isoflavone, correcting for differences in molecular weight. Results were expressed as mg of compound per 100 g on a dwb.

### 3.8. Oligosaccharides Profile of Soybean Meals

Extraction of oligosaccharides in SBM, FSBM and ESBM was performed in triplicate, according to Wongputtisin et al. [40]. Briefly, 3 g of sample was extracted with 25 mL of 50% (*v*/*v*) ethanol in an orbital shaker (300 rpm) for 1 h at 30 °C. The mixture was centrifuged at 3000× *g* for 10 min at 4 °C and the supernatant was filtered through Whatman No. 4 filter paper. Solvent was removed using a rotary evaporator at 50 °C under vacuum. The dry residue was reconstituted with 25 mL of water and filtered through a 0.45 μm PTFE filter unit before injection in the HPLC.

Analysis of oligosaccharides was performed using an HPLC system (Shimadzu, Kyoto, Japan) composed of a LC-20AT quaternary pump, a CBM-20A control system, a DGU-20A5 degasser and an LTII Evaporative Light Scattering Detector (ELSD). Chromatographic separation was achieved using a Zorbax^®^ NH_2_ column (250 × 4.6 mm, 5 μm, Santa Clara, CA, USA). The LC two-phase mobile system consisted of a gradient of water (eluent A) and acetonitrile (eluent B) at a flow rate of 1.0 mL/min. Prior to injection, the column was equilibrated with 90% solvent B. After injection of the sample, this proportion was modified to 89% solvent B after 1 min, 76% after 12 min, and 73% until the end of the 35 min run. Between injections, 10 min intervals were used to re-equilibrate the column with 90% solvent B. Identification of compounds was performed by comparison with retention time and quantification was performed by external standardization. Data were acquired by LC solution software (Shimadzu Corporation^®^, version 1.25, 2009). Results were expressed as g of compound per 100 g on a dwb.

### 3.9. Bacterial Analysis of Biscuits

Microbiological analysis was performed according to Downes and Ito [41] to investigate the presence of thermotolerant coliforms, *Salmonella* sp. and coagulase positive *Staphylococcus aureus*.

### 3.10. Sensory Acceptance of Biscuits

Sensory acceptance was carried out by students and employees of the Federal University of Rio de Janeiro (*n* = 101 at day zero; *n* = 80 after 180 days of storage). Most panelists on both days of testing were between 18 and 24 years old. On day zero, 60% of the panelists were men and 40% were women, while in the test after 180 days of storage, 48% were men and 52% were women. Biscuits samples were coded with three-digit random numbers and offered to consumers in monadic, balanced and random order. Consumers drank mineral water between the evaluation of each sample for mouth cleaning. Overall acceptability, appearance, taste, texture and aroma of biscuits were evaluated using a nine-point hedonic scale, ranging from “dislike extremely” (point 1) to “like extremely” (point 9). Purchase intention was assessed using a five-point scale, ranging from “would certainly not buy” (point 1) to “would certainly buy” (point 5). This study was approved by the ethics committee of the Federal University of Rio de Janeiro (approval number 1.110.462).

### 3.11. Statistical Analysis

Data were expressed as mean ± standard deviation of three independent experiments. Comparison of chemical composition among soybean meals, as well as among biscuits, was performed by one-way ANOVA, followed by Tukey’s test. Sensory acceptance among samples was compared by the Kruskal–Wallis test followed by Dunn’s test. Stability results of each storage time were compared to initial time using one-way ANOVA, followed by Dunnett’s test or by the Mann–Whitney test. Statistical analyses were performed using GraphPad Prism software for Windows, version 7.0 (GraphPad Software, San Diego, CA, USA). Differences were considered significant when *p* < 0.05.

## 4. Conclusions

Fermentation had a profound effect on the chemical composition of SBM, causing a positive outcome related to increases in protein, ash, dietary fiber and minerals (except for iron), removal of oligosaccharides, as well as conversion of glycosylated isoflavones to aglycones. However, biscuits produced with FSBM were not sensorially well-accepted, probably due to off flavors produced by baker’s yeast. On the other hand, enzymatic hydrolysis with commercial cellulase was able not only to decrease oligosaccharides and convert isoflavones, but also yielded biscuits with adequate sensory quality. Moreover, ESBM biscuits were chemically, microbiologically and sensorially stable when stored at room temperature for six months. Such biscuits could be labeled as dietary sources of dietary fibers, potassium, phosphorous, calcium and zinc, as well as high in proteins, copper, iron, manganese and magnesium. Two portions (60 g) of ESBM biscuits would provide 37.4 mg of isoflavones, more than the average daily amount consumed by Asian populations. Altogether, our results indicate that biscuits produced with SBM hydrolyzed with commercial cellulase have high marketing potential due to their nutritional and functional composition.

## Figures and Tables

**Table 1:** Proximate composition, minerals and oligosaccharides profile of soybean meal (SBM), fermented soybean meal (FSBM) and enzymatically-processed soybean meal (ESBM) ^1^.

	SBM	FSBM	ESBM
*Proximate composition* (g/100 g)			
Ash	6.5 ± 0.2 ^a^	7.0 ± 0.1 ^b^	6.7 ± 0.1 ^ab^
Protein	47.5 ± 1.5 ^a^	51.7 ± 0.3 ^b^	48.2 ± 0.3 ^a^
Lipid	2.2 ± 0.1 ^a^	1.9 ± 0.1 ^b^	1.5 ± 0.1 ^c^
Total dietary fiber	27.3 ± 0.5 ^a^	30.4 ± 0.2 ^b^	28.1 ± 1.0 ^ab^
Total carbohydrate	15.3	6.5	11.1
*Minerals* (mg/100 g)			
Copper	0.83 ± 0.01 ^a^	0.91 ± 0.02 ^b^	0.83 ± 0.01 ^a^
Manganese	2.4 ± 0.1 ^a^	2.6 ± 0.1 ^b^	2.4 ± 0.1 ^a^
Zinc	4.0 ± 0.1 ^a^	4.5 ± 0.1 ^b^	4.0 ± 0.1 ^a^
Iron	9.1 ± 0.2 ^a^	6.7 ± 0.1 ^b^	6.0 ± 0.2 ^c^
Sodium	13.2 ± 0.2 ^a^	18.1 ± 0.2 ^b^	12.1 ± 0.2 ^c^
Calcium	260.3 ± 2.6 ^a^	286.1 ± 1.1 ^b^	267.0 ± 5.3 ^c^
Magnesium	269.7 ± 5.7 ^a^	287.6 ± 3.5 ^b^	263.0 ± 4.5 ^a^
Phosphorus	522.0 ± 6.0 ^a^	581.8 ± 12.2 ^b^	526.1 ± 6.2 ^a^
Potassium	2046.3 ± 25.4 ^a^	2202.6 ± 44.1 ^b^	2022.9 ± 20.2 ^a^
*Oligosaccharides* (g/100 g)			
Raffinose	0.82 ± 0.05 ^a^	ND ^2^	0.43 ± 0.00 ^b^
Stachyose	2.72 ± 0.01 ^a^	ND	1.59 ± 0.02 ^b^
Sucrose	4.3 ± 0.1 ^a^	ND	2.3 ± 0.1 ^b^

^1^ Mean ± standard deviation (*n* = 3) on dry weight basis (dwb); means in the same row with different superscript letters are significantly different (ANOVA followed by Tukey’s post hoc test; *p* < 0.05). ^2^ Not detected.

**Table 2:** Contents of isoflavones and their subclasses (mg/100 g) in soybean meal (SBM), fermented soybean meal (FSBM) and enzymatically-processed soybean meal (ESBM) ^1^.

Isoflavones	SBM	FSBM	ESBM
*Subclasses*			
β-glycosides	132.4 ± 1.4 ^a^	1.2 ± 0.04 ^b^	5.6 ± 0.1 ^c^
Malonylglycosides	7.2 ± 0.1 ^a^	2.4 ± 0.2 ^b^	2.4 ± 0.04 ^b^
Acetylglycosides	9.1 ± 0.1 ^a^	0.2 ± 0.01 ^b^	2.1 ± 0.1 ^c^
Aglycones	16.0 ± 0.1 ^a^	123.8 ± 2.7 ^b^	98.1 ± 1.1 ^c^
*Total*	164.7 ± 1.8 ^a^	127.7 ± 2.9 ^b^	108.0 ± 1.3 ^c^
*Aglycones equivalents*	107.0 ± 0.7 ^a^	125.9 ± 1.9 ^b^	103.9 ± 0.9 ^a^

^1^ Values expressed as mean ± standard deviation (*n* = 3) in dry weight basis (dwb); means in the same row with superscript different letters are significantly different (ANOVA followed by Tukey’s post hoc test; *p* < 0.05).

**Table 3:** Proximate and mineral composition in one portion (30 g) of soybean meal (SBM), fermented soybean meal (FSBM) and enzymatically-processed soybean meal (ESBM) biscuits.

	SBM		FSBM		ESBM	
	Content ^1^	% DRI ^2^	Content	% DRI	Content	% DRI
Ash (g)	1.2 ± 0.1 ^b^	-	1.3 ± 0.0 ^a^	-	1.3 ± 0.1 ^a^	-
Protein (g)	7.5 ± 0.7 ^b^	13/16	8.5 ± 0.1 ^a^	15/18	8.2 ± 0.4 ^a^	15/18
Lipid (g)	4.7 ± 0.0 ^a^	-	4.4 ± 0.0 ^a^	-	4.5 ± 0.0 ^a^	-
Carbohydrate (g)	11.6	9/9	7.7	6/6	9.1	7/7
Dietary fiber (g)	4.1 ± 0.3 ^c^	11/16	6.0 ± 0.3 ^a^	16/24	4.9 ± 0.0 ^b^	13/20
Copper (mg)	0.141 ± 0.0 ^b^	16/16	0.147 ± 0.0 ^a^	16/16	0.138 ± 0.0 ^c^	15/15
Calories (kcal)	118.7	-	104.4	-	109.7	-
Iron (mg)	2.10 ± 0.1 ^a^	26/12	1.30 ± 0.0 ^c^	16/7	1.61 ± 0.0 ^b^	20/9
Potassium (mg)	361.2 ± 4.6 ^a^	11/14	366.9 ± 4.8 ^a^	11/14	344.7 ± 3.4 ^b^	10/13
Manganese (mg)	0.393 ± 0.0 ^b^	17/22	0.414 ± 0.0 ^a^	18/23	0.399 ± 0.0 ^b^	17/22
Phosphorus (mg)	103.9 ± 1.8 ^a^	15/15	110.2 ± 1.6 ^c^	16/16	106.5 ± 1.2 ^b^	15/15
Sodium (mg)	66.7 ± 0.9 ^a^	4/4	66.4 ± 1.3 ^ab^	4/4	64.8 ± 0.9 ^b^	4/4
Calcium (mg)	70.6 ± 1.0 ^a^	7/7	70.4 ± 0.8 ^a^	7/7	68.6 ± 0.5 ^b^	7/7
Magnesium (mg)	45.1 ± 1.2 ^a^	11/15	47.1 ± 0.5 ^b^	12/15	46.3 ± 1.0 ^ab^	12/15
Zinc (mg)	0.678 ± 0.0 ^a^	6/8	0.711 ± 0.0 ^b^	6/9	0.693 ± 0.0 ^ab^	6/9

^1^ Mean value of three replicates, expressed in fresh weight basis. Means in the same row with different superscript letters are significantly different (ANOVA followed by Tukey’s post hoc test; *p* < 0.05) Coefficient of variation was lower than 10%. ^2^ The percentage of the dietary recommended intake for men/women, respectively, achieved by consuming one portion of biscuits (30 g, equal to 5 or 6 units) (see DRI—Dietary Reference Intakes: https://ods.od.nih.gov/HealthInformation/Dietary_Reference_Intakes.aspx, accessed on 20 October 2022).

**Table 4:** Isoflavone profile (mg/100 g) of soybean meal (SBM), fermented soybean meal (FSBM) and enzymatically-processed soybean meal (ESBM) biscuits ^1^.

	SBM	FSBM	ESBM
*Aglycones*			
Daidzein	4.10 ± 0.21 ^a^	26.23 ± 0.69 ^b^	21.40 ± 0.26 ^c^
Glycitein	1.30 ± 0.18 ^a^	11.03 ± 0.30 ^b^	7.87 ± 0.13 ^c^
Genistein	5.93 ± 0.32 ^a^	36.30 ± 1.29 ^b^	28.72 ± 0.23 ^c^
*β* *-Glycosides*			
Daidzin	24.82 ± 1.38 ^a^	ND ^2^	0.73 ± 0.33 ^b^
Glycitin	7.83 ± 0.39 ^a^	ND	0.23 ± 0.02 ^b^
Genistin	44.24 ± 3.29 ^a^	ND	2.88 ± 0.04 ^b^
*Acetylglycosides*			
Daidzin	3.11 ± 0.29 ^a^	0.13 ± 0.00 ^b^	0.50 ± 0.01 ^b^
Glycitin	0.75 ± 0.06	ND	ND
Genistin	5.63 ± 0.30 ^a^	ND	0.85 ± 0.02 ^b^
*Malonylglycosides*			
Daidzin	1.07 ± 0.29 ^a^	1.68 ± 0.19 ^b^	1.19 ± 0.07 ^a^
Glycitin	0.15 ± 0.02 ^a^	0.35 ± 0.02 ^b^	0.10 ± 0.01 ^a^
Genistin	0.51 ± 0.08 ^a^	ND	0.18 ± 0.00 ^b^
Total isoflavones	99.43 ± 6.35 ^a^	75.72 ± 2.37 ^b^	64.64 ± 0.64 ^c^
Total aglycone equivalents	65.04 ± 4.11 ^a^	74.68 ± 2.32 ^b^	61.69 ± 0.55 ^a^

^1^ Mean ± standard deviation (*n* = 3) on dry weight basis (dwb); means in the same row with different superscript letters are significantly different (ANOVA followed by Tukey’s post hoc test; *p* < 0.05). ^2^ Not detected.

**Table 5:** Sensory acceptance and purchase intent of soybean meal (SBM), fermented soybean meal (FSBM) and enzymatically-processed soybean meal (ESBM) biscuits at day zero (*n* = 101) and after 180 days of storage (*n* = 80) ^1^.

Attributes	Day Cero			180 Days of Storage		
	SBM	FSBM	ESBM	SBM	FSBM	ESBM
Overall acceptance ^2^	5.5 ± 1.7 ^a^	3.7 ± 1.9 ^b^	5.4 ± 1.6 ^a^	5.8 ± 1.6 ^a^	3.8 ± 2.0 ^b^	5.6 ± 1.6 ^a^
Appearance ^2^	6.1 ± 1.8 ^a^	5.5 ± 2.0 ^a^	6.1 ± 1.6 ^a^	6.4 ± 1.7 ^a^	5.1 ± 1.9 ^b^	5.8 ± 1.6 ^a^
Taste ^2^	5.7 ± 1.8 ^a^	2.6 ± 1.7 ^b^	5.5 ± 1.8 ^a^	5.7 ± 1.9 ^a^	2.8 ± 2.0 ^b^	5.3 ± 2.1 ^a^
Texture ^2^	4.0 ± 1.9 ^a,b^	3.6 ± 1.9 ^a^	4.5 ± 2.0 ^b^	4.4 ± 2.0 ^a^	3.3 ± 2.0 ^b^	5.1 ± 1.9 ^a,^*
Aroma ^2^	6.3 ± 1.8 ^a^	4.8 ± 1.9 ^b^	5.3 ± 1.9 ^b^	6.5 ± 1.5 ^a^	5.1 ± 1.9 ^b^	6.3 ± 1.9 ^a,^*
Purchase intent ^3^	2.4 ± 1.0 ^a^	1.4 ± 0.7 ^b^	2.4 ± 1.0 ^a^	2.6 ± 1.1 ^a^	1.4 ± 0.8 ^b^	2.4 ± 1.0 ^a^

^1^ Data expressed as mean ± standard deviation. Superscript letters in the same row represent significant differences among biscuits formulations within a given storage time (Kruskal–Wallis test followed by Dunn’s post hoc test, *p* < 0.05). Asterisks in the same row represent significant differences between days zero and 180 within a biscuit formulation (Mann–Whitney test, *p* < 0.05). ^2^ Nine-point scale (1 = dislike extremely; 2 = dislike moderately; 3 = dislike regularly; 4 = dislike slightly; 5 = neither like nor dislike; 6 = like slightly; 7 = like regularly; 8 = like moderately; 9 = like extremely). ^3^ Five-point scale (1 = certainly would not buy; 2 = probably would not buy; 3 = maybe would buy, maybe would not buy; 4 = probably would buy; 5 = certainly would buy).

## Data Availability

The data presented in this study are available on request from the corresponding authors.

## Supplementary Materials

The following supporting information can be downloaded at: https://www.mdpi.com/article/10.3390/molecules27227975/s1, Figure S1: Aglycones (■), acetylglycosides (■), malonylglycosides (■) and β-glycosides (■) isoflavones relative content from soybean meal (SBM), fermented soybean meal (FSBM) and enzymatically-processed soybean meal (ESBM) samples; Figure S2. Chromatographic separation of isoflavones in soybean meal (SBM) (A), fermented soybean meal (FSBM) (B) and enzymatically-processed soybean meal (ESBM) (C); Table S1: Proximate (g/100 g) and mineral composition (mg/100 g) of soybean meal (SBM), fermented soybean meal (FSBM) and enzymatically-processed soybean meal (ESBM) biscuits^1^; Table S2. Microbiological stability of soybean meal (SBM), fermented soybean meal (FSBM) and enzymatically-processed soybean meal (ESBM) biscuits stored at 25 °C for 180 days; Table S3. Formulation of soybean meal (SBM), fermented soybean meal (FSBM) and enzymatically-processed soybean meal (ESBM) biscuits.

## Sample Availability

Samples are not available from the authors.
