# Supplementary material for: Biscuits Prepared with Enzymatically-Processed Soybean Meal Are Rich in Isoflavone Aglycones, Sensorially Well-Accepted and Stable during Storage for Six Months
Source: Molecules. 2022 Nov 17;27(22):7975. doi: 10.3390/molecules27227975 (PMC9699538; doi:10.3390/molecules27227975)
Supplement: Supplementary file 1 [file molecules-27-07975-s001.zip › molecules-1998252-supplementary.pdf]

molecules

Article

Biscuits Prepared with Enzymatically-Processed Soybean Meal Are Rich in Isoflavone Aglycones, Sensorially Well-Accepted and Stable during Storage for Six Months

Nathália Martins Bomfim Barreto [1], Diego Sandôra [1], Bernardo Ferreira Braz [2], Ricardo Erthal Santelli [2], Fabricio de Oliveira Silva [3], Mariana Monteiro [4,*] and Daniel Perrone [1,*]

[1] Laboratório de Bioquímica Nutricional e de Alimentos, Chemistry Institute, Federal University of Rio de Janeiro, Av. Athos da Silveira Ramos 149, CT, Bloco A, Sala 528A, Rio de Janeiro 21941-909, Brazil
[2] Laboratório de Desenvolvimento Analítico, Chemistry Institute, Federal University of Rio de Janeiro, Av. Athos da Silveira Ramos, 149, CT, Bloco A, Sala 518ª, Rio de Janeiro 21941-909, Brazil
[3] Laboratório de Desenvolvimento e Análise Sensorial de Alimentos, Faculty of Pharmacy, Federal University of Rio de Janeiro, Av. Carlos Chagas Filho, 373, CCS, Bloco A, 2nd floor, Sala 26, Rio de Janeiro 21941-902, Brazil
[4] Laboratório de Alimentos Funcionais, Nutrition Institute, Federal University of Rio de Janeiro, Av. Carlos Chagas Filho, 373, CCS, Bloco J, 2nd floor, Sala 16, Rio de Janeiro 21941-902, Brazil
* Correspondence: mariana@nutricao.ufrj.br (M.M.); danielperrone@iq.ufrj.br (D.P.)

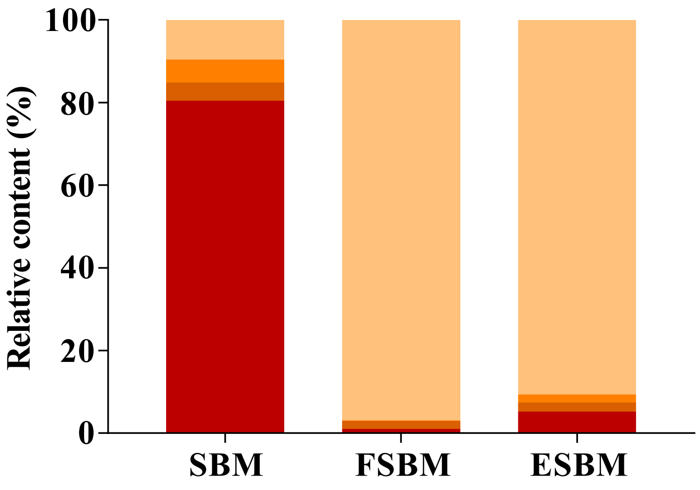

Figure S1. Aglycones ( ), acetylglycosides ( ), malonylglycosides ( ), and β-glycosides ( ) isoflavones relative content from soybean meal (SBM), fermented soybean meal (FSBM) and enzymatically-processed soybean meal (ESBM) samples.

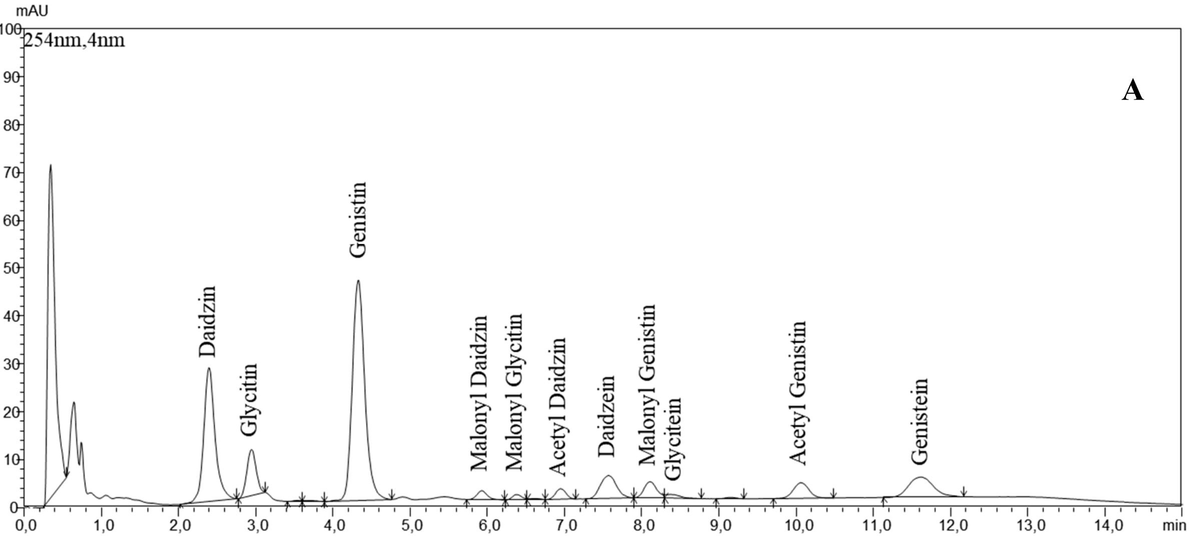
mAU
254nm,4nm
A
Daidzin
Glycitin
Genistin
Malonyl Daidzin
Malonyl Glycitin
Acetyl Daidzin
Daidzein
Malonyl Genistin
Glycitein
Acetyl Genistin
Genistein
min

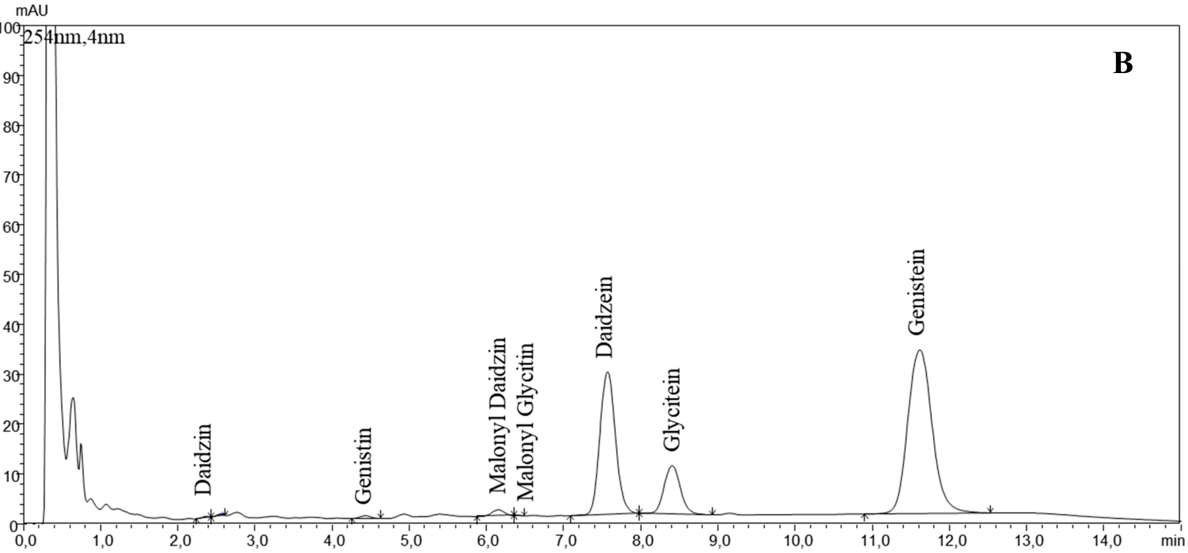
mAU
254nm,4nm
B
Daidzin
Genistin
Malonyl Daidzin
Malonyl Glycitin
Daidzein
Glycitein
Genistein
min

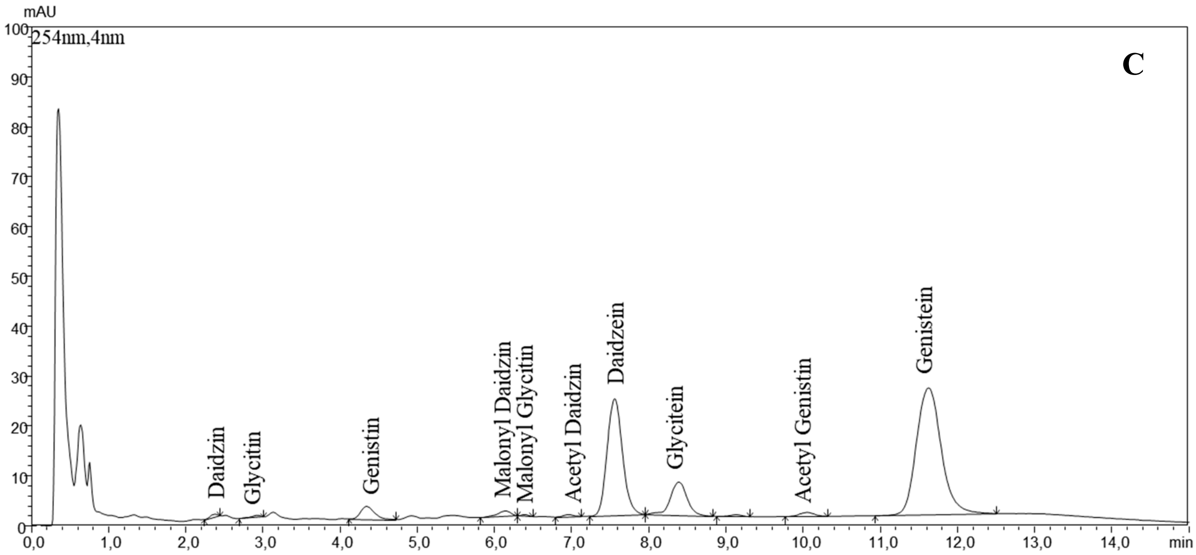

Figure S2. Chromatographic separation of isoflavones in soybean meal (SBM) (**A**), fermented soybean meal (FSBM) (**B**) and enzymatically-processed soybean meal (ESBM) (**C**).

Table S1. Proximate (g/100 g) and mineral composition (mg/100 g) of soybean meal (SBM), fermented soybean meal (FSBM) and enzymatically-processed soybean meal (ESBM) biscuits[1].

	SBM	FSBM	ESBM
Ash	4.1 ± 0.3^{a}	4.8 ± 0.1^{b}	4.6 ± 0.3^{a,b}
Protein	25.8 ± 2.3^{a}	30.3 ± 0.2^{b}	29.5 ± 1.4^{a,b}
Lipid	16.0 ± 0.01^{a}	15.9 ± 0.1^{a}	16.0 ± 0.1^{a}
Carbohydrate	36.9	20.6	25.8
Total dietary fiber	14.1 ± 1.1^{a}	21.4 ± 1.0^{b}	17.4 ± 0.2^{c}
Copper	0.5± 0.01^{a}	0.5± 0.003^{b}	0.5± 0.003^{c}
Manganese	1.3± 0.04^{a}	1.4± 0.03^{b}	1.3± 0.03^{a,b}
Zinc	2.3± 0.06^{a}	2.4± 0.04^{b}	2.3± 0.04^{a,b}
Iron	7.0± 0.2^{a}	4.3± 0.1^{b}	5.4± 0.1^{c}
Magnesium	150.4± 4.1^{a}	157.0± 1.7^{b}	154.3± 3.2^{a,b}
Sodium	222.2± 2.9^{a}	221.2± 4.2^{a,b}	216.1± 3.0^{b}
Calcium	235.2± 3.3^{a}	234.5± 2.8^{a}	228.8± 1.8^{b}
Phosphorus	346.2± 5.9^{a}	367.3± 5.5^{b}	355.1± 3.9^{c}
Potassium	1203.6± 15.5^{a}	1222.6± 15.9^{b}	1148.5± 11.5^{b}

[1] Mean ± standard deviation (n = 3) on dry weight basis (dwb); means in the same row with different superscript letters are significantly different (ANOVA followed by Tukey's post hoc test; $p < 0.05$).

Table S2. Microbiological stability of soybean meal (SBM), fermented soybean meal (FSBM) and enzymatically-processed soybean meal (ESBM) biscuits stored at 25 ^{o}C for 180 days.

Microorganism	SBM biscuit		FSBM biscuit		ESBM biscuit	
	0 days	180 days	0 days	180 days	0 days	180 days
Thermotolerant coliforms (MPN[a]/g)	< 1.0x10	< 1.0x10	< 1.0x10	< 1.0x10	< 1.0x10	< 1.0x10^{2}
Salmonella sp. (25 g)	Absence	Absence	Absence	Absence	Absence	Absence
Staphylococcus aureus (CFU[b]/g)	< 1.0x10^{2}	< 1.0x10^{2}	< 1.0x10^{2}	< 1.0x10^{2}	< 1.0x10^{2}	< 1.0x10^{2}

[a] Most probable number. [b] Colony-forming units.

Table S3. Formulation of soybean meal (SBM), fermented soybean meal (FSBM) and enzymatically-processed soybean meal (ESBM) biscuits.

Ingredient	SBM biscuit	FSBM biscuit	ESBM biscuit
Wheat flour (g)	5	5	5
SBM flour (g)	95	-	-
FSBM flour (g)	-	95	-
ESBM flour (g)	-	-	95
Table sugar (g)	30	30	30
Margarine (g)	30	30	30
Water (mL)	20	20	20
Baking powder (g)	2	2	2
Table salt (g)	1	1	1
Artificial sweetener (g)	6	6	6
Vanilla extract (mL)	10	10	10
